# Efficacy and safety of Ab interno XEN gel implant after a failed filtering surgery

**DOI:** 10.22336/rjo.2021.72

**Published:** 2021

**Authors:** Fabrizio Gaetano Saverio Franco, Marco Branchetti, Vito Spagnuolo, Marco Piergentili, Federica Serino, Maria Luce De Vitto, Enrico Bertelli, Gianni Virgili, Fabrizio Giasanti

**Affiliations:** * Careggi Hospital, Florence, Italy

**Keywords:** XEN, glaucoma, MIGS

## Abstract

**Purpose:** To evaluate the efficacy and safety of Ab interno XEN gel stent in patients with open angle glaucoma who underwent a previous failed filtering surgery.

**Methods:** A retrospective, observational, multi-centered and descriptive cohort study was conducted from January 2019 to February 2020 on patients with primary open angle glaucoma (POAG) who underwent XEN gel stent implant after a failed filtering surgical procedure. Main parameters evaluated were: changes in IOP measured with Goldmann applanation tonometer, number of anti-glaucomatous eye drops, postoperative complications, and necessity of further surgical procedures. The quantification of quality of life was obtained from the VFQ-25 questionnaire. A descriptive analysis based on pre- post- and intra-operative data was carried out.

**Results:** In all patients included in the study (6 patients for a total of 7 eyes), IOP dropped during the follow up time. We found a mean percentage reduction of -41.5% at 9 months compared to the preoperative data (baseline). IOP lowered from 21.71 ± 4.64 mmHg to 9 ± 3,21 mmHg 1 day after the operation, 11 ± 3.21 at 1 week, 11.42 ± 1.81 mmHg at 1 month, 12.42 ± 3.10 mmHg at 3 months, 13.57 ± 3.45 at 6 months, 12.71 ± 1.79 at 9 months. In 71.4%, the procedure turned out to be complication free and only one case required needling of the bleb in order to achieve optimal intraocular pressure control. A total of 71.4% patients reached a medication free regimen with a percentage of reduction in the mean number of drugs needed of -88.9%.

**Conclusion:** In our experience, XEN gel stent implant performed in Caucasian patients with POAG and a history of failed filtrating surgery, showed to be an effective and safe procedure.

## Introduction

Glaucoma is a worldwide spread optic neuropathy that leads to the progressive damage of ganglion cells followed by irreversible blindness.

The number of affected people is constantly increasing due to aging of the population with an estimated number of cases nearly around 65 million [**1**]. 

The main target of glaucoma’s treatment is lowering intraocular pressure (IOP) avoiding further damage of the optic nerve [**2**]. 

Usually, medical therapy is the first line to lower IOP, using a different type of topical molecules or laser therapy that act on different pathways of humor aqueous homeostasis.

However, since poor compliance, topical toxicity, side effects and other causes could lead to failure in controlling glaucoma progression, a surgical treatment is required in many patients [**3**,**4**]. 

Trabeculectomy and tube shunt drainage devices are currently considered the gold standard for surgical management of glaucoma, since they allow an effective reduction of IOP [**5**,**6**].

Recently, less invasive procedures commonly known as MIGS (minimally invasive glaucoma surgery) have taken root, offering a similar successful outcome, avoiding at the same time potential complications (ex. hypotony or endophthalmitis) related to traditional surgery [**7**,**8**]. 

The Xen Gel Stent (Allergan, Irvine, CA) is a collagen-based gelatin tube of 6 mm in length, with a hydrophilic nature, which creates a communication between the anterior chamber and the subconjunctival space via an *ab-interno* approach allowing the humor aqueous outflow [**9**]. 

Our study aim was to assess the efficacy and the safety of XEN gel stent surgery in patients with open-angle glaucoma (OAG), with a history of previous failed filtering surgery.

## Materials and methods

This study was designed as a cohort retrospective, observational, multi-centered, and descriptive study, involving patients affected by primary open-angle glaucoma (POAG), who had undergone XEN gel stent surgery between January 2019 and February 2020 either at the Eye Department of Careggi Hospital in Florence or at San Maurizio Regional Hospital in Bolzano.

The inclusion criteria were patients with diagnosis of POAG, with or without cataract, with a history of previous failed filtering surgery, considered as visual field (Humphrey 24-2) worsening or post-operative IOP ≥ 21 mmHg. All patients had unimpaired conjunctival mobility.

The exclusion criteria were: diagnosis of an end-stage POAG (constricted visual field less than 10 or a visual acuity of 20/200 or worse), other glaucomatous neuropathies that were not POAG, previous ocular surgeries except for phacoemulsification or filtering surgery, previous ocular trauma, progressive retinal or optic nerve diseases, corneal dystrophies.

Informant consent was obtained from all the patients undergoing surgery. The study was conducted within an ethical framework, in accordance with the Declaration of Helsinki.

The protocol included a preoperative and at 1 day, 1 week, 1, 3, 6, 9 months postoperative complete ophthalmic examination, including Snellen Best Corrected Visual Acuity (BCVA), Goldmann applanation Tonometer, biomicroscopy, gonioscopy and fundoscopy in addition with AS-OCT (MS-39, CSO, Italy) of the bleb.

Preoperatively, all the patients performed a 24-2 Humphrey visual field. 

Data concerning previous surgeries, ocular pathologies, and number of antiglaucoma drops and class was obtained from the patient’s history by accurate anamnesis and chart review.

The primary surgical outcome was the IOP reduction at the end of the follow up time. Secondary outcomes were: IOP during the follow up time, number of anti-glaucomatous eye drops at the end of the follow up, number of intraoperative or postoperative complications, necessity of a further surgical procedure to lower the IOP, patients’ quality of life.

Patients were contacted via telephone and answered the VFQ-25 questionnaire in relation to the operated eye. The answers were converted into numerical values, according to the questionnaire.

## Surgical technique

All procedures were performed by two different consultant ophthalmic surgeons (FF or PB) with a subspecialty interest in glaucoma, the same surgical technique being followed.

After topical or locoregional anesthesia, depending on the surgeon’ choice, landmarks were drawn to mark a target area of 3x3 mm in the superior-nasal conjunctiva for the XEN positioning. Then, another area for the MMC (Mitomycin C) injection site, 3 mm away from the first one, was marked in the posterior fornix. Chromatic highlighting of the XEN was then performed, to have a better view of the implant during surgery. A 27-Gauge needle was then used to inject subconjunctival 0.1 cc of MMC 0.01%. Corneal tunnels were afterwards performed and iridocorneal angle visualized with a gonio-lens. After filling the anterior chamber with viscoelastic (Healon GCV), a 27-Gauge preloaded injector was inserted through the corneal tunnel in the inferior-temporal quadrant and the stent placed under gonioscopic view superior-nasally in the ideal position. Finally, viscoelastic was withdrawn from the anterior chamber and constant irrigation with BSS was used to prime the bleb. The “priming of the bleb” allowed the checking of the function as well as the right placement of the device. Surgery was ended with hydro-suture of corneal incisions.

All patients discontinued their glaucomatous drugs on the day of surgery. Postoperative treatment included an antibiotic drop instilled 4 times a day for 1 week; a steroid drop 6 times a day for 1 week, then reducing it to 4 times a day for 3 months, 2 times a day until month 5 and once a day until month 6. 

Postoperative needling of the bleb, with subconjunctival injection of 5-fluorouracil (0.1 ml of 50 mg/ml), has been performed in case of bleb malfunctioning with high IOP values. 

## Statistical analysis

A descriptive analysis based on pre- post- and intra-operative data was carried out. For continuous variable Mean, Median, Standard Deviation, Minimum and Maximum values were chosen as parameters. In case of categorical variables, absolute and relative frequencies were reported. 

## Results

**Fig. 1:**
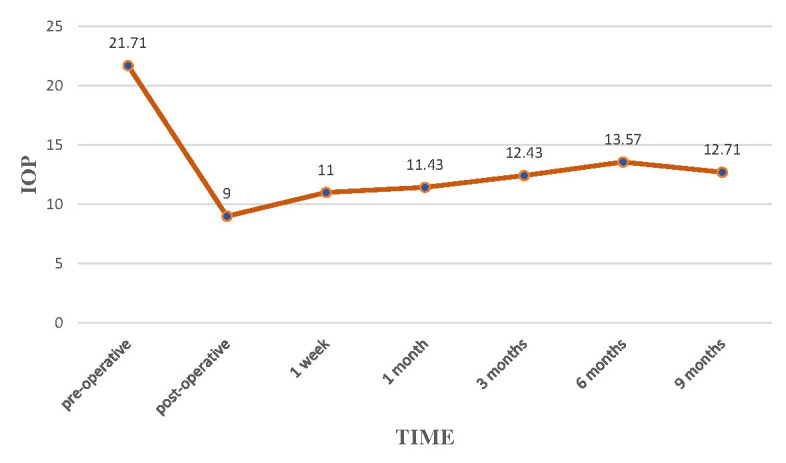
IOP evaluation during 9 months of follow-up.

A total of 7 eyes from 6 Caucasian patients were included in the study. The study population was composed of 3 males (50%) and 3 females (50%), with 4 right eyes (57.2%) and 3 left eyes (42.8%). One patient underwent surgery for both eyes. The mean age of the patient was 74.8 (range 69-89). All eyes (100%) received a previous filtering surgical procedure and cataract extraction. 5 eyes (71.4%) had a history of previous trabeculectomy, while 2 eyes of express (28.6%). 2 eyes had a myopic refractive error of more than 1.5D (28.6%), while 5 eyes were emmetropic or slightly hyperopic (71.4%). Mean preoperative IOP reduced from 21.71 ± 4.64 mmHg (range 16-30) to 9 ± 3,21 mmHg (range 4-13) on 1 day after the operation, 11 ± 3.21 (range 6-15) at 1 week, 11.42 ± 1.81 mmHg (range 11-15) at 1 month, 12.42 ± 3.10 mmHg (range 8-16) at 3 months, 13.57 ± 3.45 (range 10-20) at 6 months, 12.71 ± 1.79 (range 10-15) at 9 months. The percentage in reduction of the mean intraocular pressure at the end of the follow up (9 months) was -41.5% compared to the baseline (**Fig. 1**, **Table 1**).

**Tabel 1:** Measurement timing of IOP during 9 months of follow-up

Measurement timing of IOP (in mmHg)	Median	Mean value ± SD	Range
Pre operative data	22	21.71 ± 4.64	16-30
1 day	10	9 ± 3.21	4-13
1 week	12	11 ± 3.21	6-15
30 days	11	11.42 ± 1.81	10-15
3 months	12	12.42 ± 3.10	8-17
6 months	12	13.57 ± 3.45	10-20
9 months	13	12.71 ± 1.79	10-15

No intraoperative complications occurred (0%), while in two eyes (28.6%) we registered ocular hypotonia, respectively 1 day and 7 days after the procedure. In both cases, the refilling of the anterior chamber with viscoelastic was performed and IOP was stabilized at the normal range of values. In one case (14.3%), needling of the bleb with 5-FU 6 months after the XEN implant was performed due to a poor control of the intraocular pressure: in this patient, IOP decreased with around 5 mmHg over 3 months. 

Only two eyes (28.6%) required anti glaucomatous drugs at the end of the follow up in order to better control the IOP, while others (71.4%) were drugs free. The mean number of drugs reduced from 2.57 ± 0.79 preoperatively to 0.29 ± 0.49 at 9 months, with a percentage in reduction of -88.9%. Furthermore, no patients were treated with PG at the end of the follow up (**Table 2**).

**Tabel 2:** Number of drugs pre-operatively and number of drugs at 9 months

Patient	N° of drugs (Pre operatively)	N° of drugs (at 9 months)
1	2 (b-blocker/ carbonic anhydrase inhibitor + PG)	1 (b-blocker)
2	2 (b-blocker/ carbonic anhydrase inhibitor + PG)	0
3	2 (PG/ b-blocker + carbonic anhydrase inhibitor/ a 2-agonist)	1 (b-blocker)
4	2 (b-blocker/ carbonic anhydrase inhibitor + PG)	0
5	3 (b-blocker/ carbonic anhydrase inhibitor + PG + carbonic anhydrase inhibitor)	0
6	3 (b-blocker/ carbonic anhydrase inhibitor + PG + carbonic anhydrase inhibitor)	0
7	4 (b-blocker/ a 2-agonist + carbonic anhydrase inhibitor + PG + carbonic anhydrase inhibitor)	0

## Discussion

However, trabeculectomy augmented with MMC 0.01% remains the gold standard for the surgical management of POAG, this technique having its own limitations. 

Because of the penetrating nature of trabeculectomy as well as others filtrating procedures such as express, several serious postoperative complications may occur, such as hypotony, hyphema, flat anterior chamber, choroidal detachment, choroidal effusion or hemorrhage, endophthalmitis, and surgery-induced cataract if performed in phakic eye [**10**]. 

Apart from those range of complications, trabeculectomy surgery is limited by a suboptimal long-term success rate. Rate of failure has been reported to be between 23% to 51% at 5 years [**11**,**12**]. 

Episcleral or subconjunctival fibrosis are thought to be the main reason of failure. In case of a failed trabeculectomy, subsequent surgeries include a second trabeculectomy or placement of a tube shunt.

Nowadays, MIGS (minimally invasive glaucoma surgery) represents an effective and less invasive alternative to filtering procedures. 

In our study, we described the outcomes of a cohort of 7 eyes that underwent XEN stent implant augmented with MMC after a previous failed filtering surgery, in term of efficacy and safety.

It is worth noting that the IOP at the end of the follow up time dropped to around 41.5% from the baseline (preoperative) remaining stable over time. These results are in line with literature: Aitor Fernandez Garcìa et al. reported a decrease in IOP of around 25% in a cohort of 93 eyes affected by POAG, which underwent XEN gel implant and were followed up for 36 months. Similar results were shown by De Gregorio’s study, in which IOP lowered with 40%. Karimi at al. evaluated 17 eyes with POAG, which have undergone XEN gel stent after a failed trabeculectomy and obtained an IOP reduction of 37% [**13**-**15**]. 

Other important data our study revealed, was a reduction in the number of anti-glaucomatous drugs after the surgery. At the end of the follow up, 71.4% of the patients were drugs free, with no patients requiring PG, leading to a better compliance, and at the same time avoiding ocular discomfort. In fact, glaucoma drugs used to be related to a widespread number of side effects, which include conjunctival inflammation (especially PG), allergy, ocular surface toxicity, skin pigmentation, etc. [**16**]. 

In addition to VFQ-25 questionnaire results, this demonstrates an improvement in the patient’s quality of life. 

The Tube vs. Trabeculectomy Study, designed to evaluate the safety and efficacy of Baerveldt-350 tube shunt implant compared to trabeculectomy with MMC in eyes with prior cataract or trabeculectomy surgery, confirmed the relative efficacy and safety of shunt devices in those cases. Postoperative complications were reported in 29% of the patients belonging to the tube group, while in 41% of the patients from the trabeculectomy group, a variable percentage in success between 40 and 80% was registered [**17**,**18**]. 

Post operative anti-inflammatory therapy with use of intensive corticosteroids was used to prevent bleb fibrosis, which represent the main cause of shunt failure. With this protocol in only one eye (14.3%), the needling of the bleb under topical anesthesia with subconjunctival injection of 5-fluorouracil (0.1 ml of 50 mg/ml) was performed in order to control IOP. Needling rate is reported to be variable in literature. In their retrospective study, Tan et al. showed a percentage of 51.3% for eyes requiring bleb intervention, while Jeremy et al. reported a high needling rate of around 62% during one year follow up in a cohort of East Asian eyes, which underwent XEN gel implantation. The reason for such variable needling rate may be related to the fact that bleb correct functioning is influenced by multiple factors. 

Bleb needling is a safe procedure but not free from complications, such as hypotony, endophthalmitis, suprachoroidal hemorrhage, etc., for this reason, avoiding bleb intervention means a lower rate of such complications [**19**]. 

Furthermore, in our study population only two complications (28.6%) occurred, showing a good profile of safety. No bleeding was described, while in both cases ocular hypotony rapidly resolved, filling the anterior chamber with viscoelastic.

The rate of early postoperative hypotony in our study was acceptable but higher than other studies such as the tube vs. trabeculectomy in which this complication is described in 10% of the cases [**20**]. 

It is possible that in our cases cumulative exposure to MMC from a previous filtering surgery might have altered the conjunctiva and the ciliary body functioning leading to a higher rate of postsurgical hypotony compared to XEN gel stent performed as a single procedure [**21**,**22**]. 

Limitations of this study include the fact that was retrospective and descriptive, the limited number of patients included and the short term follow up might have underestimated long term complications or failure. A prospective study with a larger number of eyes and a longer follow up should be carried out in order to confirm and reinforce these results. 

## Conclusion

Based on the results of our study we can assert that XEN gel stent implant is a safe surgical procedure in the glaucoma management with low intraoperative complications and only two cases of hypotony reported (28.6%) that rapidly returned to the normal range with anterior chamber viscoelastic fulfill. In addition, no further surgeries were required following the XEN implant and only in one eye the needling of the bleb was performed in order to control the IOP.

Furthermore, comparing pre and post operative data we found a great effectiveness of the procedure with a constant reduction in IOP that remained stable during the follow up time. Also, the number of drugs needed to obtain a satisfying reduced IOP led to a better patient’s quality of life with less ocular discomfort and side effects related to anti glaucomatous eye drops, especially PG. In conclusion, we can affirm that XEN gel stent implant is a safe and effective procedure in the treatment of patients affected by POAG after a failed filtering procedure such as express or trabeculectomy. 

**Conflict of Interest statement**

Authors state no conflict of interest.

**Informed Consent and Human and Animal Rights statement**

Informed consent has been obtained from all individuals included in this study.

**Authorization for the use of human subjects**

Ethical approval: The research related to human use complies with all the relevant national regulations, institutional policies, is in accordance with the tenets of the Helsinki Declaration, and has been approved by the review board of Careggi Hospital, Florence, Italy.

**Acknowledgements**

None.

**Sources of Funding**

None.

**Disclosures**

None.
